# Heightened Procoagulation after Post-Operative Thromboprophylaxis Completion in Patients with Metastatic Bone Disease from Primary Colorectal Cancer

**DOI:** 10.3390/jcm11247397

**Published:** 2022-12-13

**Authors:** Lisa Yamaura, Daniel Young, Leslie Skeith, Michael J. Monument, Craig N. Jenne, Antoine Dufour, Prism Schneider, Ejaife O. Agbani

**Affiliations:** 1 McCaig Institute for Bone and Joint Health, Cumming School of Medicine, University of Calgary, Calgary, AB T2N 4N1, Canada; 2 Department of Surgery, Cumming School of Medicine, University of Calgary, Calgary, AB T2N 4N1, Canada; 3 Libin Cardiovascular Institute, Cumming School of Medicine, University of Calgary, Calgary, AB T2N 4N1, Canada; 4 Department of Medicine, Division of Hematology and Hematological Malignancies, Cumming School of Medicine, University of Calgary, Calgary, AB T2N 4N1, Canada; 5 Arnie Charbonneau Cancer Institute, Cumming School of Medicine, University of Calgary, Calgary, AB T2N 4N1, Canada; 6 Department of Microbiology, Immunology and Infectious Diseases, Cumming School of Medicine, University of Calgary, Calgary, AB T2N 4N1, Canada; 7 Department of Critical Care Medicine, Cumming School of Medicine, University of Calgary, Calgary, AB T2N 4N1, Canada; 8 Department of Biochemistry and Molecular Biology, Cumming School of Medicine, University of Calgary, Calgary, AB T2N 4N1, Canada; 9 Department of Physiology and Pharmacology, Cumming School of Medicine, University of Calgary, Calgary, AB T2N 4N1, Canada

**Keywords:** platelet procoagulant membrane dynamics (PMD), venous thromboembolism, metastatic bone disease, colorectal cancer, orthopaedic surgery

## Abstract

Background: Platelets play a role in venous thromboembolism (VTE) and in mediating colorectal cancer (CRC) progression. Still, platelets’ role in hypercoagulability after surgical intervention for metastatic bone disease (MBD) is ill-defined. Methods: In this quantitative observational study, we utilized a high-resolution imaging approach to temporally examine platelet procoagulant membrane dynamics (PMD) in four patients with MBD from primary CRC (CRC/MBD), before and after surgical intervention, over a 6-month period. We coupled this investigation with thrombelastography, quantitative plasma shotgun proteomics, and biochemical analysis. Results: The plasma of CRC/MBD patients was enriched in ADAM1a, ADAMTS7, and physiological ligands for platelet glycoprotein-VI/spleen tyrosine kinase (GPVI/Syk) activation. Thromboprophylaxis attenuated procoagulation upon its initial prescription (post-operative day one, POD1); however, all patients experienced rebound procoagulation between POD3 and POD14, which was associated with Syk activation (Y525/Y526) in all patients, and a VTE event in two patients. Plasma levels of DNA-histone complexes increased steadily after surgery and remained elevated throughout the study period. Additionally, we increasingly sighted both homotypic and heterotypic platelet microaggregates after surgery in CRC/MBD patients, but not in healthy control participants’ plasma. Conclusions: Our data elucidates the cell biology of a prothrombo-inflammatory state caused by disease and vascular injury, and recalcitrant to thromboprophylaxis. New mechanistic insights into hypercoagulability in CRC/MBD patients may identify novel drug targets for effective thromboprophylaxis type and duration after orthopaedic surgery.

## 1. Background

Colorectal cancer (CRC) is the fourth most commonly diagnosed cancer and the third leading cause of cancer-related death worldwide [1]. Up to 90% of cancer-related deaths are attributed to metastasis [2], and the systemic spread of CRC occurs at the early stages of the disease, with metastatic seeding preceding clinical detection of CRC [3]. CRC often spreads to bone (metastatic bone disease, MBD) occurring between three months and four years after initial diagnosis [4]. Due to the presence of bone lesions which destroy (lytic) or deposit (blastic) bone predominantly in weight-bearing bones (e.g., proximal femur), the development of MBD is associated with increased risk for pathologic fracture [5]. These fractures often necessitate orthopaedic surgery for prophylactic stabilization or acute fixation. Malignancy and orthopaedic surgery are independent risk factors for venous thromboembolism (VTE) [6], which encompasses deep vein thrombosis (DVT) and potentially life-threatening pulmonary embolism (PE). Despite the clinical significance of VTE prevention, clearly defined thromboprophylaxis guidelines for patients with MBD undergoing surgical fixation of bone metastases are unavailable, and no consensus exists on the optimal type or duration of thromboprophylaxis for these patients. Whilst the American Society of Hematology (ASH) guidelines for lower extremity orthopaedic surgery recommend beginning thromboprophylaxis 12 h post-operatively and extending this for up to 42 days post-operatively [7], Thrombosis Canada recommends thromboprophylaxis for 14–35 days [8].

Hypercoagulability is associated with malignancy, and VTE risk is 7-fold higher in patients with MBD [9]. Platelet releasates play a role in VTE pathogenesis and in the mediation of CRC progression [10]. In addition, platelet-tumour cell interactions can promote metastasis. Still, platelets’ role in hypercoagulability after surgical intervention for MBD is ill-defined. Here, we utilized detailed dynamic imaging to visualize platelets of patients with MBD which had developed from primary CRC (henceforth referred to as CRC/MBD patients), before and after surgical intervention. Over a 6-month period, we characterized platelet activation and procoagulation mechanisms through the systematic analysis of platelet procoagulant membrane dynamics (PMD). We coupled this investigation with thrombelastography (TEG), quantitative plasma proteomic analysis, and biochemical studies. We report plasma enriched in physiological ligands for platelet glycoprotein (GP) VI activation, in tandem with platelet and neutrophil procoagulation, which continued beyond the duration of post-operative thromboprophylaxis in CRC/MBD patients. New mechanistic insights into hypercoagulability in CRC/MBD patients may identify novel drug targets for effective thromboprophylaxis type and duration after orthopaedic surgery.

## 2. Methods

### 2.1. Study Participants and Design

**Figure 1:**
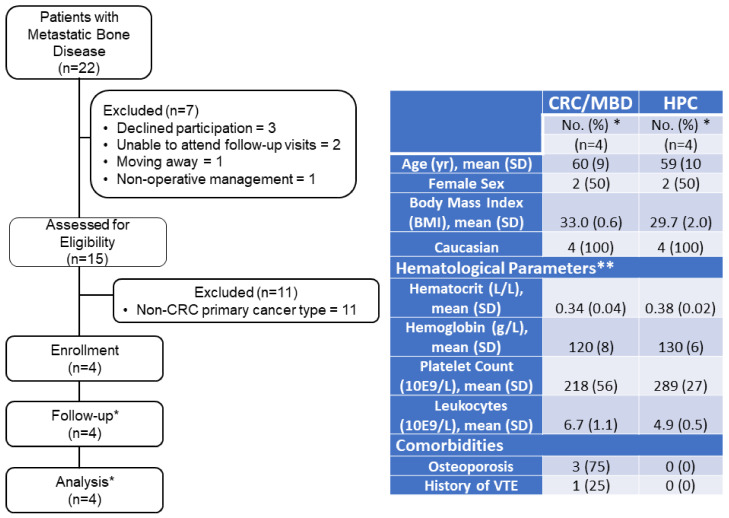
Flowchart of study recruitment and Participant Demographics and Anthropometric Data. * Unless noted otherwise; ** pre-operative values in CRC/MBD patients.

Adults (≥18 years) who presented to Foothills Medical Centre (Calgary, AB) with primary colorectal cancer which had metastasized to bone, and who required orthopaedic surgery to treat acute or impending pathologic fractures, were consecutively enrolled into this single-centre, observational prospective cohort study. In addition to bone metastases, all patients had visceral metastasis to the liver, lung, or both. Patients who were pregnant, receiving treatment for acute VTE, or who had primary bone tumours were excluded from participation. None of the patients had a history of long-term antiplatelet use prior to enrollment, nor were any patients using antiplatelet agents during the study. Participants were studied between December 2020 and September 2022 (Figure 1). During the 6-month study period, blood samples were collected at eight timepoints: pre-operatively; on post-operative days (POD) one, three, and five while the patient was admitted to hospital; and at follow-up visits 14-, 42-, 90-, and 180 days post-operatively. Sterile venipuncture was performed to collect whole blood samples in 2.7 mL vacutainer tubes with 3.2% sodium citrate additive for TEG analysis, PMD analysis, quantitative shotgun proteomics, and biochemical analysis. On POD3, patients underwent a bilateral lower extremity Doppler ultrasound (LEDUS) for DVT screening. Patients were prescribed thromboprophylaxis at the discretion of the treating orthopaedic surgeon. Throughout the study, VTE incidence and thromboprophylaxis prescription and adherence were captured via medical chart pulls during the patient’s hospital stay, and post-discharge, via patient interview during follow-up visits. Incidence of VTE was recorded if VTE events were image-confirmed (i.e., LEDUS for DVT; CT PE or VQ Scan for PE) and interpreted by a radiologist blinded to the study protocol and outcome measures. Accordingly, VTE events were categorized as asymptomatic (i.e., discovered during DVT screening) or symptomatic. Control samples were obtained from age- and sex-matched healthy participant controls (HPC).

### 2.2. Thrombelastography (TEG)

TEG analysis was performed by pipetting whole blood (1 mL) into a TEG global microfluidic cartridge compatible with the TEG^®^6s haemostasis analyzer (Haemonetics Corporation, Boston, MA, USA), as previously described [11,12].

### 2.3. Platelet-Rich Plasma Preparation

Whole blood was centrifuged at 180× *g* for 17 min to prepare platelet-rich plasma (PRP). All blood samples were processed within 1–2 h of collection. Given the higher density of preformed platelet microaggregates, platelet heterotypical aggregates, and degranulated leukocytes, we aimed to visualize these structures in PRP+ [13]. PRP+ was prepared by centrifuging whole blood at 180× *g* for 17 min, followed by a careful extraction of the supernatant, which included the upper platelet-rich-plasma (PRP) fraction and the buffy coat [13].

### 2.4. Biochemical Analysis

Enzyme-linked immunosorbent assay (ELISA) experiments were performed according to the manufacturer’s specifications (Cell Death Detection Kit; Roche, Basel, Switzerland), and Western blot analysis was performed as previously reported [14]. Human Phospho-SYK (Y525/Y526) and Direct-Blot™ HRP anti-β-actin antibodies were from R & D Systems (MAB6459, Minneapolis, MN, USA) and BioLegend (664803, San Diego, CA, USA), respectively.

### 2.5. Immunoblotting

Washed platelets were prepared as previously described [15] from patients’ PRP at timepoints indicated. Proteins were separated by electrophoresis using 8–15% Tris glycine polyacrylamide gels against known molecular weight markers and transferred onto PVDF membranes. After blocking with 5% BSA in Tris-buffered saline/Tween-20 (10 mM Tris, 150 mM NaCl, and 0.1% Tween 20), membranes were probed with the appropriate primary and horseradish peroxidase-conjugated secondary antibodies, and proteins were detected by enhanced chemiluminescence.

### 2.6. Quantitative Proteomics

Plasma and platelet samples from HPC and CRC/MBD patients were subjected to quantitative shotgun proteomics analysis, as previously described [16]. Samples were prepared by the filter-assisted separation of peptides (FASP) method. Briefly, 100 µg of total protein was precipitated by adding trichloroacetic acid (TCA) to a final concentration of 50%, followed by incubation on ice. Samples were centrifuged at 14,000× *g* for 15 min at 4 °C. Samples were washed three times in 100% ice cold acetone and stored at −20 °C. Samples were resuspended in 8 M tris-urea solution by shaking for 20 min. To reduce disulfide bonds, 10 mM of dithiothreitol (DTT) was added at 37 °C for 30 min. Carbamidomethyl modification of the cystines was completed by adding 50 mM of iodoacetamide (IAA) at room temperature in the dark. Samples were moved to the top of a 30 kDa filter (sigma catalog no: MRCF0R030), washed by adding 100 µL of wash solution to the top of the filter, and then centrifuged at 14,000× *g* for 15 min. Samples were washed three times with 8 M tris-urea, followed by three times with 50 mM ammonium bicarbonate. Samples were trypsinized at 37 °C overnight at a ratio of 1:10 trypsin: total protein, and subsequently, eluted off the filter membrane by washing with 50 mM ammonium bicarbonate. Finally, samples were subjected to c18 clean up with waters solid-phase extraction (SPE) column (Catalog: WAT020525) according to the manufacturer’s directions before injection on the orbitrap mass spectrometer.

### 2.7. Platelet Procoagulant Membrane Dynamics (PMD) Imaging

**Figure 2:**
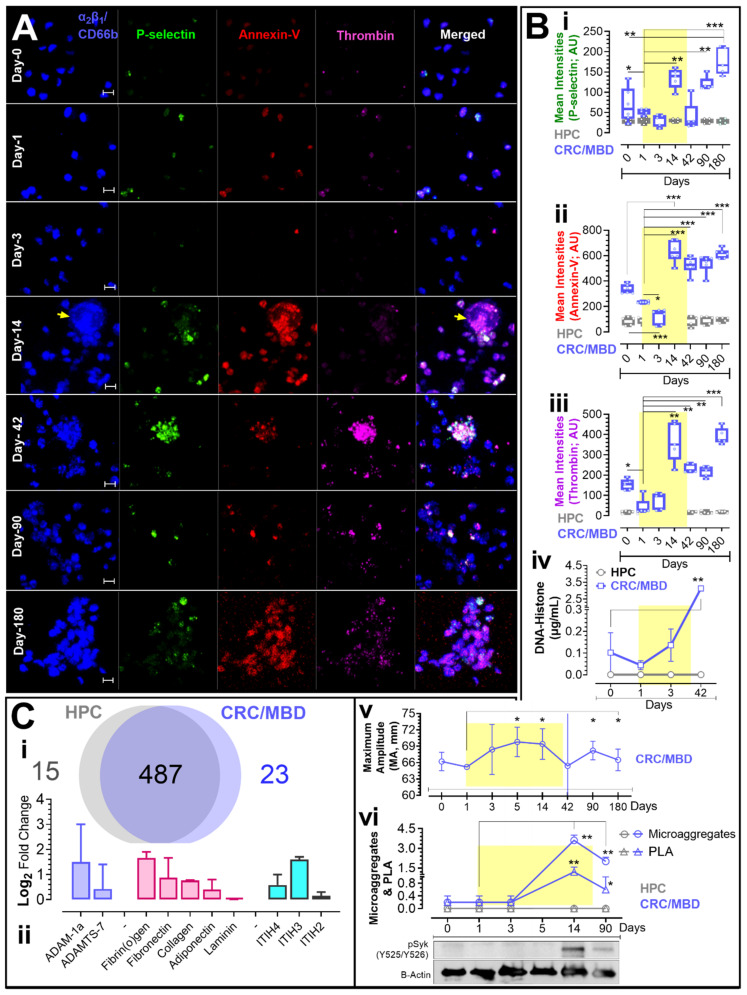
Platelet Procoagulant Membrane Dynamics and Plasma Proteomics Studies in Patients with Metastatic Bone Disease from Primary Colorectal Cancer (CRC/MBD) Before and After Surgery. Citrated platelet-rich plasma (PRP) was recalcified to 1 mM and allowed to adhere to bovine serum albumin (BSA)-coated surfaces. (**A**): Extended focus images were captured at the 45 min timepoint. Images in column 1–4 (from the left-hand side) display fluorescent markers of platelet collagen receptor integrin ⍺2β1 and CD66 (AlexaFluor^®^ 405 conjugated, blue), P-selectin expression (Alexa-fluor^®^ 488 anti-human CD62P antibody, green), phosphatidylserine (PS) exposure as indicated by AlexaFluor^®^ 568 annexin-V, (red) binding, and thrombin generation on the platelet membrane (AlexaFluor^®^ 647 anti-thrombin, magenta). The fifth column is a merged view. (**Bi**–**Biii**): Mean fluorescent signal intensities of pooled experiments are summarized in boxplots for: (**i**) P-selectin expression, (**ii**) PS exposure, and (**iii**) thrombin generation in CRC/MBD patients (blue) and healthy participant controls (HPC) (grey). (**Biv**): Enzyme-linked immunosorbent assay (ELISA) results for circulating DNA-Histone complexes. (**Bv**): Plot of serial thrombelastography (TEG) of whole blood from CRC/MBD patients. (**Bvi**): Temporal changes in mean microaggregate count and mean platelet-leukocyte aggregate (PLA) count. Image shows Western blot probe for spleen tyrosine kinase (phospho-Syk @Y525/526) in CRC/MBD platelet lysates at the indicated timepoints, and corresponding beta actin. The yellow underlay in (**Bi**–**Bvi**), denotes the period of standard thromboprophylaxis care. (**C**): Quantitative shotgun proteomics analysis showing (**Ci**) proteins upregulated in CRC/MBD plasma and (**Cii**) Log2-fold change in physiological glycoprotein VI ligands and thrombo-inflammatory markers. Data were from four CRC/MBD patients and four HPC. Data were tested for normality using the Shapiro–Wilk test (*p* < 0.05). Data were then analyzed using GraphPad Prism 9.5 (San Diego, CA, USA) and presented as box-and-whiskers plots showing minimum to maximum values. The dots/points in the chart represent raw data (replicate experiments inclusive). The medians and interquartile ranges of data are represented by the horizontal line through the box and height of the box, respectively. Statistical significance was determined by 1-way ANOVA and Bonferroni post hoc test. *p* < 0.05 (*) or *p* < 0.01 (**) or *p* < 0.001 (***) were considered significant. Images were captured at Nyquist using Nikon A1R laser scanning confocal microscope (original objective magnification, ×60) and analyzed using Volocity^®^ Software (Quorum Technologies). Scale bars: 3 μm (**A**).

Fluorescent image acquisition and analysis were done as previously reported [13,14,15,16,17,18]. Briefly, aliquots of platelet rich plasma (PRP) recalcified to 1 mM with calcium chloride (CaCl_2_) were preincubated with fluorescent probes (as indicated, Figure 2) and added onto MatTek dishes pre-coated with bovine serum albumin (BSA). Adherent plasma platelets were fixed with 4% paraformaldehyde for 15 min after 45 min of incubation. Platelets were labelled for collagen receptor integrin ⍺_2_β_1_ and CD66 (Alexa-Fluor^®^ 405 conjugated, blue), P-selectin expression (Alexa-Fluor^®^ 488 anti-human CD62P antibody green), phosphatidylserine (PS) exposure (Alexa-Fluor^®^ 568 annexin-V red), and membrane thrombin formation (AlexaFluor^®^ 647 anti-thrombin magenta). High-resolution 3D fluorescent images of cells adherent over BSA-coated surfaces were obtained at 25 °C using a Nikon A1R confocal microscope. Image resolution was improved by the restoration complement of Volocity^®^ imaging Software Suite 6.5 and analyzed using the same software (Quorum Technologies Inc., Puslinch, ON, Canada).

### 2.8. Statistical Analysis

Statistics were performed using Prism 9.5 (GraphPad Software, San Diego, CA, USA), and individual analyses are reported in the Figure 2 legend. Significant change in protein expression from the proteomics data was determined as the outliers after boxplot R analysis. Outliers were determined according to Tukey’s definition [19].

## 3. Results

Four CRC/MBD patients and four healthy participant controls (HPC) were enrolled into this study (Figure 1). Patients were prescribed low molecular weight heparin (LMWH) for 28 days post-operatively, except one patient with prior DVT history, who resumed apixaban (5 mg, twice daily) use post-operatively. VTE rate was 50% in this cohort, with two patients experiencing acute asymptomatic but clinically significant proximal DVT during the 6-month study period. DVT was confirmed in one patient on POD3 (treated with apixaban, 10 mg, twice daily for five days) and in the other patient on POD5 (treated with weight-based dosing of LMWH at 175 units/kg, once daily for eight days). Both patients were prescribed ongoing thromboprophylaxis (apixaban, 5 mg, twice daily) after acute DVT treatment and remained on this medication throughout the study period. In addition, both patients with VTE outcomes were also being treated for non-insulin-dependent diabetes mellitus and hypertension. One of the patients had tumour debulking and cemented total hip replacement with cage reconstruction surgery, and the other underwent cephalomedullary nailing.

### 3.1. Procoagulant Membrane Dynamics, Biochemical Analysis & Thrombelastography

Our systematic analysis of platelet PMD indicated that platelets in all four CRC/MBD patients circulated in an extensively preactivated state when compared to HPC platelets (Figure 2A,B). Platelet preactivation (Figure 2A,(Bi)) and procoagulation (Figure 2A,(Bii,Biii)) were evident pre-operatively, which increased further after completion of thromboprophylaxis. We determined platelet preactivation through increased platelet surface expression of P-selectin, an adhesion molecule, and confirmed procoagulation by the resultant annexin-V binding after membrane phosphatidylserine (PS) externalization, and by thrombin formation on the platelet membrane (Figure 2A,B) [13,14,15,17,18,20]. Notably, post-operative thromboprophylaxis attenuated hypercoagulation in all patients between POD1 and POD5; however, in all CRC/MBD patients, there was rebound procoagulation between POD5 and POD14, which was associated with a VTE event in two patients. Results of assessment of plasma levels of DNA-histone complexes (Figure 2(Biv)) and thrombelastography (Figure 2(Bv)) followed the procoagulation pattern revealed by our PMD studies. DNA-histone level increased steadily after surgery and remained elevated throughout the study period (Figure 2(Biv)). Both homotypic and heterotypic platelet aggregates were increasingly sighted after surgery in our CRC/MBD patients when compared to HPC (Figure 2(Bvi)).

### 3.2. Western Blot and Proteomic Analysis

Platelets of CRC/MBD, but not HPC showed increased spleen tyrosine kinase (Syk) phosphorylation at Tyr-525/Tyr-526 on POD14 and POD90 (Figure 2(Bvi)). In addition, our POD90 shotgun quantitative proteomic analysis showed increased plasma levels of two proteases, ADAM1a and ADAMTS7, of the adamalysins family in CRC/MBD patients compared to HPC (Figure 2C). Proteomic analysis also revealed plasma enriched in fibrinogen (alpha, beta chains; FGA, FGB), fibronectin (FN1, anastellin), collagen (alpha-1(V, XIV)) chains, and adiponectin (ADIPOQ) (Figure 2C). The thrombo-inflammatory mediators inter-⍺-trypsin inhibitor heavy chain proteins (ITIH2, ITIH3, and ITIH4) were likewise enriched in plasma from CRC/MBD patients, but not HPC (Figure 2C). Our unbiased proteomic approach enabled us to profile multiple peptides of the identified proteins. We noted two unique peptides for ADAM1a and three unique peptides for ADAMTS7. In addition, we provide additional information about the biological state of these peptides in vivo. We identified the ADAM1a peptides to be acetylated, and for ADAMTS7, two peptides were acetylated and one was deamidated.

## 4. Discussion

DNA-histone level increased steadily after surgery and throughout the study period, consistent with the procoagulation pattern revealed by PMD studies. This was potentially indicative of increasing neutrophil extracellular trap (NETs) formation, which is known to contribute to a thrombo-inflammatory state in multiple disease states [21,22,23]. Though distinct and driven by separate mechanisms [24], both homotypic and heterotypic platelet aggregation increased after surgery. The appearance of these structures further confirmed the cell biology of a prothrombo-inflammatory state caused by both disease and vascular injury, and recalcitrant to thromboprophylaxis. Most antiplatelet agents in current clinical use target mediators of platelet activation, and homotypic but not heterotypic aggregation [25]. Likely, the lack of suitable antiplatelet agents or combination of agents targeting thrombosis and homotypic aggregation pathways, coupled with the formation of NETs and heterotypic aggregates, may account for the heightened procoagulation in our CRC/MBD patients during and after completion of thromboprophylaxis. Additionally, it is possible that disease progression and cancer treatment may also have contributed to the prolonged hypercoagulability observed in these patients. Post-operatively, patients often undergo adjuvant radiation therapy, and over time, extent of malignancy may progress. After day 90, all patients received radiation therapy (mean: 26 ± 6 Gy in 6 ± 1 sessions). In addition to radiation therapy, two patients received chemotherapy from study day 90 to day 180. One patient was treated with capecitabine (2000 mg daily) and another received lanreotide injections (120 mg) every four weeks. Cancer progression occurred in two patients between study day 90 and day 180, and all our CRC/MBD patients received radiation therapy at 3 months post-operation.

Proteomic analysis showed increased plasma levels of ADAM1a and ADAMTS7 proteases in CRC/MBD patients compared to HPC. This aligns with previous studies demonstrating essential roles for adamalysins in CRC etiopathogenesis and metastasis, and the potential for adamalysins to be sensitive predictors and monitors of CRC progression and targetable mediators of the disease [26]. Proteomic analysis also revealed plasma enriched in fibrinogen, collagen chains, and adiponectin, which are all proteins previously shown to activate platelets via the GPVI receptor and to trigger intravascular thrombosis [27,28]. This enrichment may be due to the actions of ADAMTS7, a metalloprotease, able to cleave, remodel, and degrade the vascular extracellular matrix [29]. Consistent with previous findings after GPVI activation [27], platelets of CRC/MBD but not HPC showed increased Syk phosphorylation and POD14 and beyond. In addition, the inhibition of GPVI was shown to attenuate platelet–tumour cell interaction and tumour metastasis [30]. Syk activation has been reported to regulate integrin signalling in neutrophils and platelets, and to increase platelet-platelet interactions and intravascular thrombosis [27,28]. Syk activation via GPVI may account for the heightened procoagulation and VTE incidence reported in CRC/MBD patients observed in this study. Interestingly, studies have now demonstrated that the platelet GPVI/Syk activation may also promote metastasis through interaction with cancer cell-derived galectin-3 [30]. Data on the role of ITIH proteins in CRC is limited; however, these proteins may function to preserve the elevated risk for thromboembolic complications in these patients [31]. Taken together, plasma levels of procoagulant agonists, such as fibronectin, or of DNA-histone complexes, may be practical markers to evaluate risk of thrombosis in CRC/MBD patients.

The lack of additional control groups is a limitation to the generalizability of our results as we are unable to define the extent of the contribution of CRC/MBD alone, or the orthopaedic surgical intervention alone, to the hypercoagulable state of our study participants. As CRC/MBD is associated with extensive disease burden and limited life expectancy, coupled with recruitment occurring at a single centre, enrollment for this study was challenging and a small sample size is a limitation of this brief report. However, within this cohort, observations were consistent and validated across multiple methods of analysis. A larger-scale study would provide opportunity to both corroborate and leverage the findings of this study to elucidate platelet-cancer cell interactions and to identify novel drug targets for effective thromboprophylaxis in CRC/MBD patients.

## 5. Conclusions

Overall, our findings demonstrate that CRC/MBD patients are hypercoagulable for a period extending beyond the typical timeframe for post-operative thromboprophylaxis. It is likely that the hypercoagulable state of our CRC/MBD patients was, in part, platelet and leukocyte driven (through the activation of phosphoSyk-Y^525^/Y^526^) and perpetuated by plasma enrichment with procoagulant agonists and the ITIH family of thrombo-inflammatory mediators.

## Data Availability

The proteomics data are publicly available and were deposited in PRIDE Archives, accession number: PXD034160. (Username: reviewer_pxd034160@ebi.ac.uk Password: nAwkmdt5). The R codes are available upon request.

## Abbreviations

ASH	American Society of Hematology
BSA	Bovine serum albumin
CaCl_2_	Calcium chloride
CRC	Colorectal cancer
DVT	Deep vein thrombosis
DTT	Dithiothreitol
ELISA	Enzyme-linked immunosorbent assay
FASP	Filter-assisted separation of peptides
GP	Glycoprotein
HPC	Healthy participant controls
ITIH	Inter-⍺-trypsin inhibitor heavy chain proteins
IAA	Iodoacetamide
LMWH	Low molecular weight heparin
LEDUS	Lower extremity Doppler ultrasound
MBD	Metastatic bone disease
NETs	Neutrophil extracellular traps
VTE	Venous thromboembolism
CRC/MBD	Patients with metastatic bone disease from primary colorectal cancer
PS	Phosphatidylserine
PRP	Platelet-rich plasma
POD	Post-operative day
PMD	Procoagulant membrane dynamics
PE	Pulmonary embolism
SPE	Solid-phase extraction
Syk	Spleen tyrosine kinase
TEG	Thrombelastography
TCA	Trichloroacetic acid
PRP+	Upper plasma-platelet fraction and buffy coat
